# Human β-Defensin 2 Expression in Oral Epithelium: Potential Therapeutic Targets in Oral Lichen Planus

**DOI:** 10.3390/ijms20071780

**Published:** 2019-04-10

**Authors:** Abdelhakim Salem, Rabeia Almahmoudi, Jaana Hagström, Holger Stark, Dan Nordström, Tuula Salo, Kari K. Eklund

**Affiliations:** 1 Department of Clinical Medicine, Clinicum, University of Helsinki, 00014 Helsinki, Finland; 2 Translational Immunology Research Program, University of Helsinki, 00014 Helsinki, Finland; tuula.salo@helsinki.fi (T.S.); kari.eklund@helsinki.fi (K.K.E.); 3 Department of Oral and Maxillofacial Diseases, Clinicum, University of Helsinki, 00014 Helsinki, Finland; rabeia.mustafa@helsinki.fi; 4 Department of Pathology, Helsinki University Hospital, Helsinki, Finland and Translational Cancer Medicine Research Program, Faculty of Medicine, University of Helsinki, 00014 Helsinki, Finland; jaana.hagstrom@helsinki.fi; 5 Institute of Pharmaceutical and Medicinal Chemistry, Heinrich Heine University Düsseldorf, 40225 Düsseldorf, Germany; stark@hhu.de; 6 Department of Internal Medicine, Helsinki University and Helsinki Hospital, 00014 Helsinki, Finland; dan.nordstrom@hus.fi; 7 Medical Research Centre, Oulu University Hospital, 90220 Oulu, Finland; 8 Cancer and Translational Medicine Research Unit, University of Oulu, FI-90014 Oulu, Finland; 9 Department of Rheumatology, Helsinki University and Helsinki University Hospital, and Orton Orthopedic Hospital and Research Institute, 00014 Helsinki, Finland

**Keywords:** histamine, histamine H4 receptor, human beta defensin 2, mast cells, inflammation, bacterial lipopolysaccharide (LPS), OPMDs, oral epithelium, oral cancer, oral tongue squamous cell carcinoma

## Abstract

Human β-defensin 2 (hBD-2) is a potent antimicrobial peptide that participates in defense against invading bacteria. We recently showed that bacterial components and histamine, through histamine H4 receptor (H4R), are involved in the pathogenesis of the potentially malignant lesion, oral lichen planus (OLP). However, the underlying mechanisms remain unknown. We, therefore, investigated the role of hBD2–histamine crosstalk signaling in promoting OLP pathology. Biopsies from OLP and oral tongue squamous cell carcinoma (OTSCC) patients, and healthy controls were used. Two OTSCC cell lines and normal human oral keratinocytes (HOKs) were used. HBD-2 and other targets were mapped by immunostaining and analyzed by ImageJ2 software. The highly sensitive droplet-digital PCR technology and qRT-PCR were utilized to study the clinically derived and in vitro samples, respectively. H4R was challenged with the specific agonist HST-10 and inverse agonist ST-1007. HBD-2 was highly induced in OLP lesions. In contrast, hBD2 expression was attenuated in OTSCC tissues, while very low levels of hBD-2 messenger RNA (mRNA) were observed in OTSCC cells. Together with tumor necrosis factor-α (TNF-α), histamine upregulated hBD-2 mRNA expression in HOKs. Activation of H4R seems to modulate the expression of epithelial hBD-2. These findings suggest the involvement of hBD-2 in the pathogenesis of OLP and may, thus, be harnessed for therapeutic interventions in OLP.

## 1. Introduction

Human β-defensin 2 (hBD-2) is a potent antimicrobial peptide produced by epithelial cells, which has a key role in mounting innate immune response against bacteria and other microbial infections [1,2]. The expression and production of hBD-2 are induced mainly by bacterial endotoxins (lipopolysaccharides, LPS) and proinflammatory cytokines such as tumor necrosis factor-α (TNF-α), interferon-γ (IFN-γ), and interleukin-1β (IL-1β) [1]. In spite of its role in combating pathogens, altered regulation of hBD-2 is associated with various disorders including cancer [3]. Oral cancer is one of the most common cancers in the world, affecting, inter alia, the oral mucosa and tongue [4]. Importantly, oral tongue squamous cell carcinoma (OTSCC) accounts for more than 50% of all malignant tumors in the oral cavity [5]. Despite the recent advances in cancer therapy, the prognosis and survival rate of OTSCC patients remain rather poor due to the high risk of occult lymph node metastasis and cancer-related mortality [6,7]. In fact, the progressive transformation of normal epithelium to OTSCC is a multistep process that comprises a complex interaction between environmental and inflammatory mediators [8,9,10,11]. Inflammatory processes can facilitate cancer development by enhancing immune cell infiltration and stromal remodeling [12]. Oral potentially malignant disorders (OPMDs), including some chronic inflammatory lesions such as oral lichen planus (OLP), predispose patients to develop OTSCC [13,14,15]. Indeed, a better understanding of the molecular mechanisms involved in such tumorigenic process will pave the way for developing novel and more effective drugs for OTSCC.

Histamine is a potent pleiotropic mediator that is widely distributed throughout the body tissues. We recently reported that bacterial LPS was associated with altered histamine metabolism in OLP [16]. Such dysregulated production of histamine inhibited epithelial adhesion proteins and downregulated histamine H4 receptor (H4R) in oral epithelium [16,17]. Furthermore, we showed that low H4R expression, together with increased mast cell (MC) count, was correlated with poorly differentiated OTSCC cells [18]. However, the underlying mechanisms behind these observations are still elusive. Interestingly, hBD-2 level was downregulated in oral cancer patients and accompanied by increased bacterial infections [19]. Likewise, downregulation of hBD-2 level was also observed in cervical, thyroid, and esophageal cancer cells [20,21]. Hence, the aim of our study was to investigate whether hBD-2, as an essential epithelial peptide induced by LPS, plays a role in the OLP pathogenesis cascade and whether histamine can modulate such a process. In addition, based on our findings and recent reports, we aim to envision a potential scenario of the inflammatory cascades that may precede an OLP-mediated OTSCC. Here, we used clinical biopsies obtained from OLP and OTSCC patients, and we studied the in vitro cell signaling using normal human oral epithelial cells and two OTSCC-derived invasive cell lines.

## 2. Results

### 2.1. High Expression of hBD-2 in OLP Lesions

**Figure 1:**
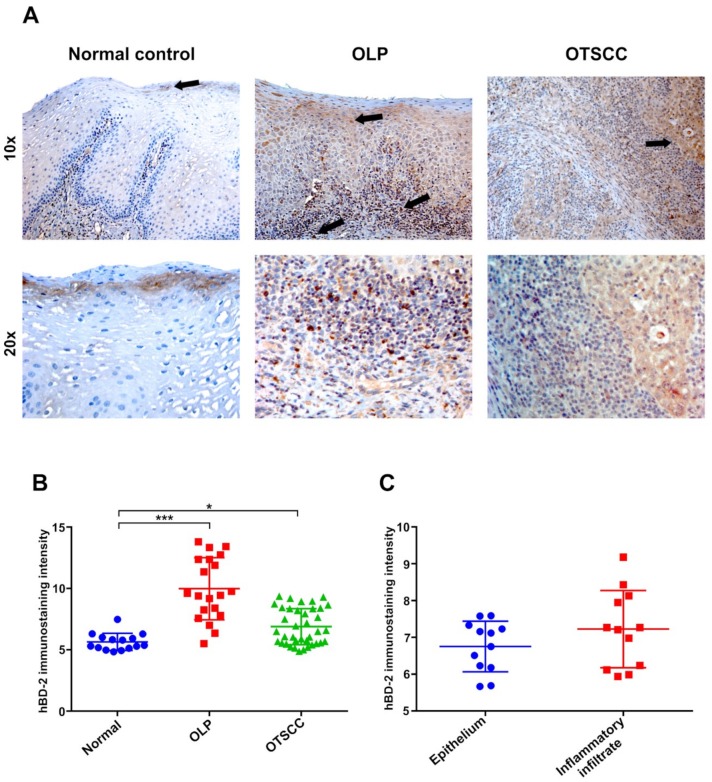
(**A**) Faint staining of human β-defensin 2 (hBD-2) in normal oral epithelium, which is more confined in the uppermost superficial layers (black arrow). Oral lichen planus (OLP) lesions exhibited strong epithelial and subepithelial hBD-2 expression. (**B**) In oral tongue squamous cell carcinoma (OTSCC) specimens, hBD-2 was also induced, but the staining was generally less intense than in OLP. (**C**) HBD-2 is equally expressed in the epithelial and subepithelial regions in OLP lesions. Data are expressed as means ± SD; *n* = 14, 14, and 40 for controls, OLP, and OTSCC, respectively; * *p* ≤ 0.05, *** *p* ≤ 0.001. Kruskal–Wallis test followed by Dunn’s multiple comparisons correction test (**B**) and Mann–Whitney U test (**C**) were applied.

**Figure 2:**
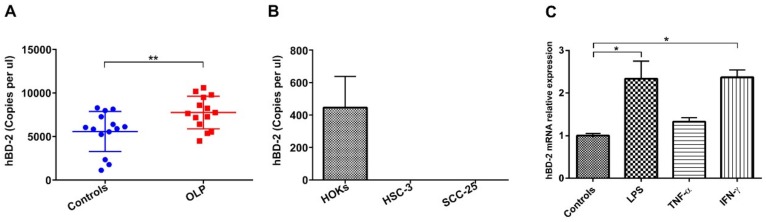
(**A**) Absolute quantification by droplet-digital (dd)PCR technology revealed a substantial upregulation of hBD-2 levels in OLP patients (average of ~8000 copies/µL) compared with normal individuals. (**B**) On the contrary, hBD-2 was robustly downregulated in tongue cancer cells (HSC-3 and SCC-25) compared with the highly expressed hBD-2 in normal human oral keratinocytes (HOKs). (**C**) Stimulating HOKs with bacterial lipopolysaccharide (LPS) and interferon-γ (IFN-γ) for 8 h induced higher hBD-2 levels compared with tumor necrosis factor-α (TNF-α) and non-stimulated control cells. Data are expressed as means ± SD; *n* = 14 for controls and OLP; * *p* ≤ 0.05, ** *p* ≤ 0.01. Mann–Whitney U test (**A**) and Kruskal–Wallis test followed by Dunn’s multiple comparisons correction test (**C**) were applied.

To determine the expression of hBD-2 protein in oral epithelium, immunohistochemical analysis was performed on patient and control tissue samples. In healthy specimens, hBD-2 staining was either negative or faintly positive in superficial epithelial cells, with a few hBD-2+ immune cells in the lamina propria. In contrast, OLP lesions showed higher immunoreactivity of hBD-2 in all the epithelial layers, as well as in the subepithelial inflammatory cell infiltrate (Figure 1A). Interestingly, hBD-2 was also induced in OTSCC specimens as compared with healthy controls (Figure 1A). Overall, hBD-2 was more intensively expressed in OLP lesions and OTSCC than in normal controls (*p* = 0.001 and 0.05, respectively; Figure 1B). Further analysis was performed on OLP specimens, which revealed an induced immunoreactivity of hBD-2 in the subepithelial inflammatory infiltrates (Figure 1C). Next, the highly sensitive droplet-digital (dd)PCR was utilized to quantify hBD-2 messenger RNA (mRNA) level in clinical samples from OLP patients (*n* = 14) and normal controls (*n* = 14). Both normal and OLP tissues expressed hBD-2 mRNA; however, hBD-2 was more strongly induced in OLP specimens compared to controls, with an average of ~8000 copies/µL of patient sample (*p* = 0.01; Figure 2A). In comparison with the high expression in human oral keratinocytes (HOKs), the hBD-2 mRNA was not detected in HSC-3 nor in SCC-25 cells (Figure 2B).

### 2.2. LPS and Inflammatory Mediators Upregulate hBD-2 Level in Oral Epithelial Cells

We recently reported that proinflammatory mediators, such as histamine, cytokines, and bacterial LPS, play a role in the pathogenesis of OLP and OTSCC [16,17,18]. Thus, the aim was to determine whether hBD-2 expression in oral epithelial cells is modulated by these mediators. Indeed, we found that both LPS and IFN-γ significantly (*p* = 0.03 and 0.02, respectively) induced the expression of hBD-2 mRNA in HOKs compared with the controls. TNF-α alone slightly, though not significantly, upregulated hBD-2 mRNA (Figure 2C).

### 2.3. Epithelial Cells and MCs Are Major Sources of hBD-2 in OLP

**Figure 3:**
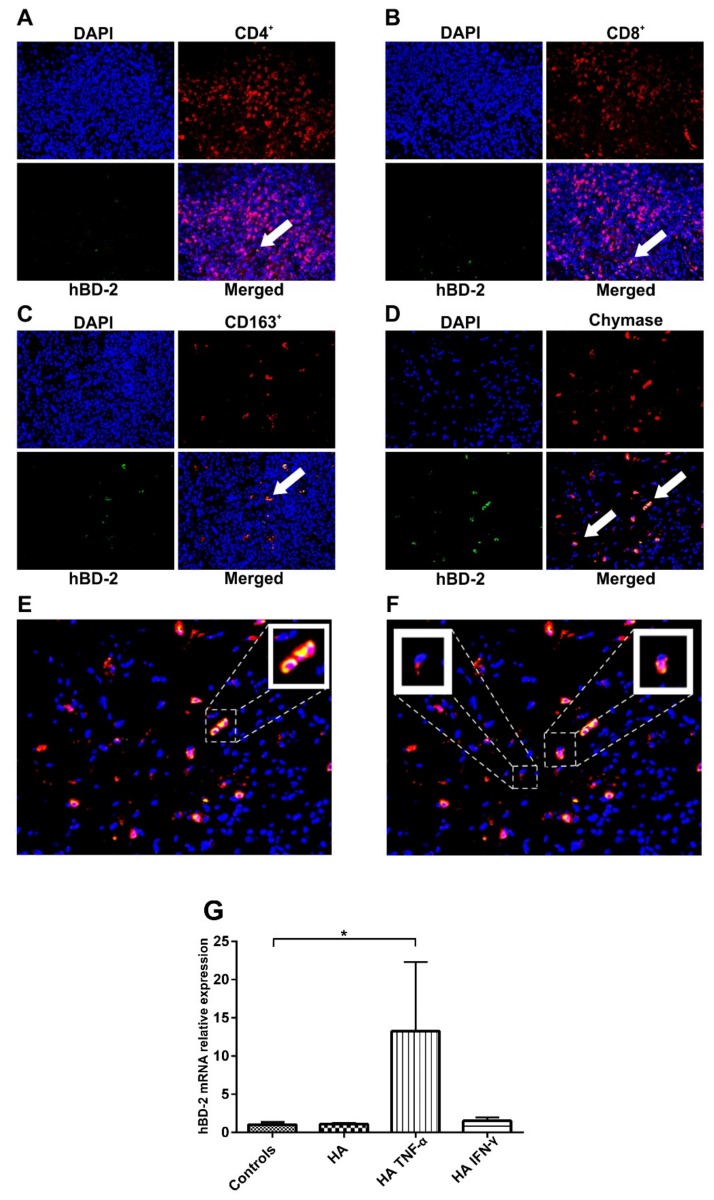
(**A**,**B**) The subepithelial inflammatory cell infiltrate was mapped by double-label immunofluorescence staining, which revealed low hBD-2-immunoreactivity (green) in cluster of differentiation 4-positive (CD4+) and CD8+ (red) T-lymphocytes. (**C**,**D**) On contrary, CD163+ (red) macrophages showed more hBD-2+ cells, while most of the chymase+ (red) mast cells (MCs) were hBD-2+; white arrows indicate hBD2+ cells. (**E**,**F**) Non-degranulated MCs were strongly positive for hBD-2 compared with the degranulated cells. Magnifications = 10× (**A**–**D**), 20× (**E**,**F**), and 40× (small boxes). (**G**) Histamine alone did not have a significant effect on the hBD-2 level; however, histamine strongly promoted TNF-α-mediated, but not IFN-γ-mediated, upregulation of hBD-2 mRNA. Data are expressed as means ± SD; * *p* ≤ 0.05. Kruskal–Wallis test followed by Dunn’s multiple comparisons correction test was applied.

Immunostaining revealed that hBD-2 was expressed in OLP samples in epithelial cells and lamina propria alike; hence, we investigated the plausible “subepithelial” sources of hBD-2 in OLP lesions. The subepithelial cell infiltrate was mapped with double-label immunofluorescence staining. Very low immunoreactivity of hBD-2 (green) was observed in cluster of differentiation 4-positive (CD4+) and CD8+ (red) T lymphocytes (Figure 3A,B; white arrows indicate hBD2+ cells), while CD163+ macrophages (red) were moderately positive (Figure 3C). However, most of the subepithelial MCs (red) were hBD-2+ (Figure 3D). HBD-2 staining appeared to be stronger in intact “non-degranulated” MCs than in the degranulated cells (Figure 3E,F, respectively). To further elucidate the role of MCs in the regulation of hBD-2 production by oral epithelial cells, we examined the effect of histamine and TNF-α, both produced by MCs, on hBD-2 production in HOKs. Having shown that TNF-α alone did not significantly alter the hBD-2 level (Figure 2C), it was, therefore, interesting that histamine together with TNF-α, but not with IFN-γ, induced the expression of hBD-2 (*p* = 0.01; Figure 3G).

### 2.4. Attenuated H4R Immunoreactivity in OLP Lesions

**Figure 4:**
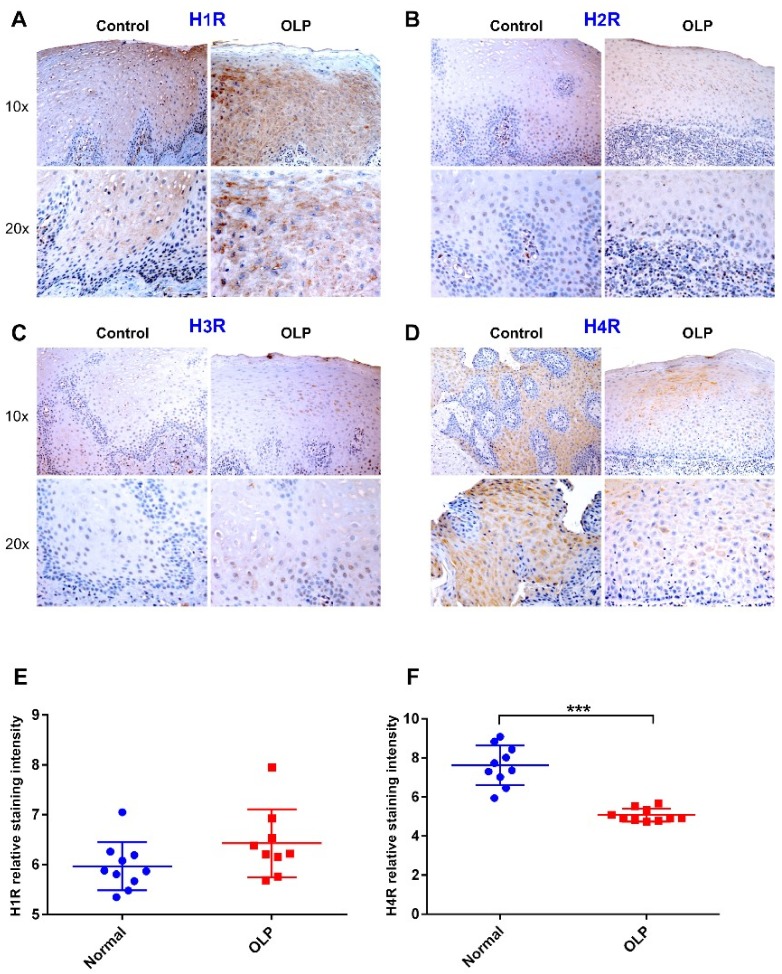
(**A**) Immunostaining assays showed strong expression of histamine receptor 1 (H1R) in normal epithelium and in OLP lesions. (**B**,**C**) H2R and H3R staining were very faint in both normal and OLP specimens. (**D**) In contrast, H4R was strongly expressed in normal controls compared with the markedly weakened expression in OLP lesions. The staining shows that H1R and H4R were expressed mainly in the cytoplasm and cell membranes of epithelial cells. (**E**,**F**) ImageJ analysis demonstrated almost equal expression of H1R in control and OLP samples, while expression of H4R was very low in OLP lesions. Data are expressed as means ± SD; *n* = 10 for controls and OLP groups; *** *p* ≤ 0.0001. Mann–Whitney U test was applied.

Histamine signals via four G-protein-coupled receptors (H1R through H4R). Hence, we next examined the receptor subtypes involved in histamine-mediated enhancement of hBD-2 production by oral epithelial cells. Immunohistochemical staining revealed that H1R was expressed in normal and OLP epithelium, with a slightly more prominent immunoreactivity in OLP specimens, although the difference was not statistically significant (Figure 4A,E). H2R and H3R staining revealed very faint or negative expression in both OLP lesions and normal epithelium (Figure 4B,C). In contrast, H4R was strongly expressed in all normal epithelial layers, but it was markedly lower in OLP lesions where the uniform staining pattern was lost and shifted toward the upper epithelial layers (*p* < 0.0001; Figure 4D,F). H1R and H4R were expressed mainly in the cytoplasm and cell membranes of epithelial cells.

### 2.5. Role of H4R in TNFα- and LPS-Mediated hBD-2 Expression

**Figure 5:**
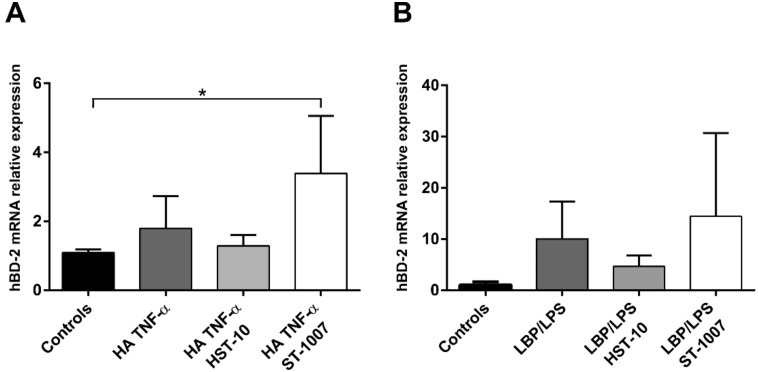
(**A**) Activation of H4R with its agonist HST-10 slightly decreased hBD-2 expression induced by histamine (HA) and TNF-α as compared with the controls (8 h), while this effect was reversed by the inverse agonist ST-1007. (**B**) In a similar trend, ST-1007 induced hBD-2 expression in HOKs via a lipopolysaccharide-binding protein (LBP)/LPS-mediated pathway (24 h), whereas HST-10 diminished such an effect. Data are expressed as means ± SD; * *p* ≤ 0.05. Kruskal–Wallis test followed by Dunn’s multiple comparisons correction test was applied.

We showed in a previous study that H4R was able to provide cell survival signals which protect against apoptosis in human salivary cells [22]; thus, we asked whether H4R is also crucial for the regulation of hBD-2 signaling in oral epithelium. To unravel this question, HOKs were firstly incubated for 8 h with H4R ligands (H4R-specific agonist HST-10, and inverse agonist ST-1007) and then stimulated with inflammatory mediators (for 8 h) and bacterial LPS (for 24 h) as relevant factors for OLP pathogenesis [16]. Interestingly, ST1007-activated HOKs showed an upregulation of the histamine/TNF-α-induced hBD-2 activity compared with controls (*p* = 0.04). On contrary, this effect seems to be reversed by the receptor agonist, HST-10, which showed a moderate, although not statistically different, downregulation of hBD-2 via the same histamine/TNF-α-mediated pathway (Figure 5A). Likewise, ST-1007 induced hBD-2 expression in HOKs stimulated with lipopolysaccharide-binding protein (LBP)/LPS for 24 h (*p* = 0.06), while HST-10 seems to lessen such effect in a similar trend observed in the histamine/TNF-α-mediated pathway; however, these effects were not statistically significant (Figure 5B).

## 3. Discussion

Oral epithelium serves as the first line of defense against microbial invasion. One of the key defense mechanisms of epithelium is the secretion of anti-microbial peptides [1,2]. In OPMDs, such as OLP, the defensive barrier function is impaired due to the disruption of epithelial integrity, thus rendering the epithelium more permeable to pathogens [23,24,25]. We previously reported that histamine is involved in the loss of epithelial integrity in OLP [16]. Under such circumstances, microbial pathogens and bacterial toxins can persistently penetrate to deeper oral epithelial cells, bind to pattern-recognition receptors, induce potent inflammatory responses and stimulate hBD-2 production [26,27,28].

The hBD-2 was shown to induce direct anti-microbial immune response and mediate immune cell chemotaxis (e.g., T lymphocytes and macrophages) and enhance their cytokine production [29]. Synthesis and production of hBD-2 by epithelial cells are significantly induced by inflammatory mediators including TNF-α, IFN-γ, and IL-1β [28,30]. Of note, persistent production of such proinflammatory mediators was associated with pro-carcinogenic “smoldering” inflammation that promotes tumorigenesis [31,32,33]. In this study, we observed higher expression of hBD-2 in OLP and tongue cancer lesions as compared with normal oral epithelium. Importantly, tumor-derived cells had markedly low transcriptional levels of hBD-2. This suggests a potential association between hBD-2 and the chronic inflammatory (and tumorigenic?) process in OLP. These findings were further confirmed by determining the exact copy numbers of the hBD-2 mRNA, which was obtained using the highly-sensitive ddPCR technology. These studies also revealed considerably increased hBD-2 copies in OLP patients, compared with the healthy controls, and marked loss of hBD-2 in OTSCC cell lines. Of note, our findings are in agreement with previous studies showing significantly lower basal hBD-2 mRNA expression in samples obtained from oral cancer patients [34,35].

In fact, hBD-2 upregulation in OLP could in part be enhanced by bacterial invasion through the impaired epithelial barrier. Choi et al. showed that OLP lesions exhibit substantially increased levels of penetrating bacteria, which were observed in the epithelial layers, as well as in the subepithelial T cell infiltrate, and were positively correlated with the lesional intensity [25]. Interestingly, one of the most common bacterial species in OLP lesions, which also mediates hBD-2 signaling, *Porphyromonas gingivalis*, was recently linked to oral carcinogenesis via enhancing epithelial–mesenchymal transition [9,36,37,38]. In accordance with these reports, we demonstrated that hBD-2 was induced by ultrapure bacterial LPS, suggesting a role for Gram-negative bacteria in hBD-2-mediated pathogenesis of OLP. In addition to bacterial penetration, proinflammatory mediators from subepithelial cell infiltrate could also contribute to hBD-2 overexpression in OLP lesions. We demonstrated here that hBD-2 expression was equally intense in the epithelial and subepithelial (lamina propria) regions of OLP lesions.

MCs are multifunctional immune cells that play crucial roles in tumor development and progression [39]. We recently reported that the MC-derived mediator, IL-17F, might be harnessed as a reliable prognostic factor in early-stage OTSCC patients [40]. Furthermore, we showed that MCs and their major granular constituent, histamine, are increased in OPMDs and could probably be involved in oral carcinogenesis [18]. In addition, histamine was able to disrupt epithelial integrity by inhibiting integrin-α6 and integrin-β4, while supernatants from activated human MCs (i.e., MC-releasate) markedly induced oncogene expression in oral epithelium [16,18]. Moreover, MC-releasate downregulated the expression of H4R, which plays a key role in orchestrating inflammatory responses and antitumor immunity [41,42]. Here, we demonstrate that MCs may represent a major source of hBD-2, which signifies their role in the dysregulated antimicrobial response in OLP. However, the expression of hBD-2 by MCs does not ascertain that they also secrete the protein, which should be further confirmed by other approaches. Nevertheless, MCs are probably involved in hBD-2-mediated pathogenesis by releasing histamine, which is able to stimulate hBD-2 production from human keratinocytes [43]. Importantly, epithelial-derived hBD-2 can in turn induce Ca2+ mobilization and degranulation in MCs, via Mas-related gene X2 (MrgX2), and stimulate release of histamine, which leads to further production of hBD-2 [44,45].

**Figure 6:**
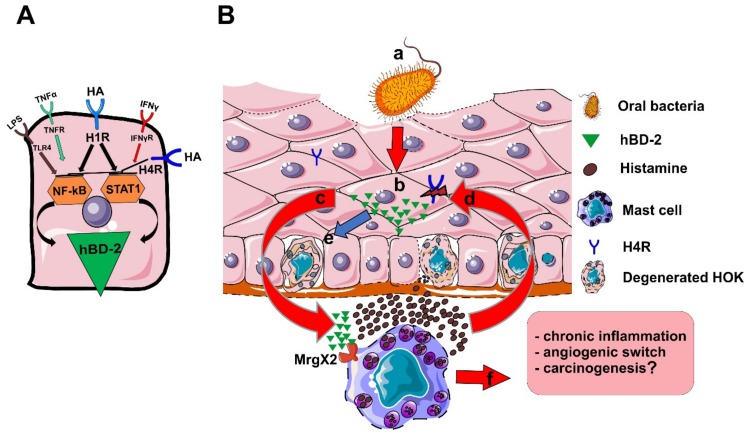
(**A**) The effect of histamine (HA) on hBD-2 production is likely mediated via nuclear factor-κB (NF-κB) and, to lesser extent, via signal transducer and activator of transcription 1 (STAT1); hBD-2 promoter region contains two sites for NF-κB and one site for STAT1, which are both induced by histamine signaling. Effect of H4R on hBD-2 in HOKs could be mediated via both STAT1 and NF-κB axes. (**B**) The following scenario is proposed to illustrate the role of histamine-BD-2 axis in perpetuation chronicity in OLP: a) bacterial invasion into deeper layer cells is enhanced by the impaired barrier integrity in OLP; b) epithelial cells secrete considerable amounts of hBD-2; c) hBD-2 from epithelial cells stimulates mast cell (MC) degranulation and histamine release via Mas-related gene X2 (MrgX2); d) histamine, in turn, activates oral epithelial cells to produce more hBD-2 and, thus, perpetuates a proinflammatory vicious circle; e) Surplus histamine and hBD-2 may result in further MC degranulation, degeneration of the epithelial cell barrier, and loss of H4R; f) MC degranulation enhances the proinflammatory response by recruiting more immune cells and driving angiogenesis (angiogenic switch), which may partly promote carcinogenesis in OLP lesions.

The synergistic effect of histamine with TNF-α on hBD-2 production is likely mediated through the increased expression of transcription factors, as the promoter region of hBD-2 contains two binding sites for nuclear factor-κB (NF-κB) and one site for signal transducer and activator of transcription 1 (STAT1) [46]. However, it is not clear why histamine alone did not alter hBD-2 levels, although it was previously shown to activate NF-κB through H1R [47]. Interestingly, activation of H4R showed regulatory properties on hBD-2 expression in cultured oral epithelial cells. H4R agonist interfered with the histamine/TNF-α- and LPS-mediated signaling pathways, whereas the inverse agonist, ST-1007, showed a similar trend by enhancing their effect, although some of these effects were not statistically significant. In this regard, activation of H4R was able to suppress both STAT1- and TNF-α-mediated effects in different cell lines [22,48]; thus, it is conceivable to assume that such an HST10-mediated effect could also be caused by interfering with one or both pathways (Figure 6A). Supporting this assumption, H4R expression was noticeably low in OPMDs and cancer, which could partly explain the dysregulated production of hBD-2 in such disorders [18].

Overall, and based on the published reports and our findings, the following scenario could be envisioned, at least theoretically, to illustrate the role of histamine-BD-2 axis in perpetuation of the persistent inflammation in OLP (Figure 6B): (a) the impaired barrier integrity in oral epithelium facilitates bacterial invasion into deeper epithelial layers; (b) oral epithelial cells respond by secreting hBD-2; (c) epithelial-derived hBD-2 induces MC degranulation and histamine release via MrgX2; (d) histamine in turn activates epithelial cells to produce more hBD-2 and, thus, perpetuates a proinflammatory vicious cycle; (e) elevated levels of surplus histamine and hBD-2 can promote further degeneration of the epithelial cell barrier and loss of inhibitory H4R [16]; (f) MC degranulation enhances the proinflammatory response by recruiting more immune cells and driving angiogenesis (angiogenic switch), which overall promotes cancer development in OPMDs [49].

In conclusion, we suggest that hBD-2 expression is persistently dysregulated in the potentially malignant OLP lesions. There is now an overwhelming evidence, from cell lines to clinical trials, supporting the role of histamine receptors in inflammation-mediated cancer development, which may contribute to the identification of novel targets for cancer treatment [50]. Importantly, present findings suggest potential regulatory properties of H4R ligands in oral epithelium and support previous reports that H4R agonists may impart promising outcomes in chronic inflammatory lesions and their potential development to cancer [50,51]. However, since some results obtained from H4R-activated samples and controls were not statistically significant, and due to the lack of further controls, it remains difficult to draw a solid conclusion from the presented data about the participation of H4R in regulating hBD-2 production in HOKs. Nevertheless, our findings raise at least the possibility of such an association between H4R and hBD-2 in oral epithelium; thus, further functional studies are warranted. Moreover, we acknowledge some limitations in our study, such as a limited amount of functional experiments and a lack of in vivo assays. The development of effective and safe molecules for cancer prevention and therapy is crucial. Therefore, further studies on the role of H4R ligands and whether they can be harnessed as therapeutic targets in OPMDs to abate their potential progression to cancer are needed.

## 4. Materials and Methods

### 4.1. Patient Samples and Ethical Approval

This study included a total of 68 biopsied samples of oral mucosal tissues that were obtained from healthy controls (*n* = 14), OLP patients (*n* = 14), and OTSCC patients (*n* = 40). Control samples were collected during wisdom tooth extraction. OLP samples were obtained from the department of pathology at Helsinki University hospital. OTSCC samples were obtained from Oulu University hospital (ethical authorization numbers and details are below). Biopsies were taken from active lesion areas by a specialized oral surgeon under local anesthesia (20 mg/mL xylocaine and 12.5 µg/mL adrenaline) and fixed in 10% neutral buffered formalin, dehydrated in an increasing ethanol series, cleared in xylene, and embedded in paraffin (FFPE). The Ethics Committee of the Hospital District of Helsinki and Uusimaa approved the study (authorization number: 42/13/03/01/2013; date: 20 February 2013; location: Helsinki, Finland). The usage of OTSCC patient materials was approved by the Ethical Committee of the Northern Ostrobothnia Hospital District, Finland (49/2010, 56/2010) and the Finnish National Supervisory Authority for Welfare and Health (6865/05.01.00.06/2010). All participants gave their written informed consent prior to participating in this study.

### 4.2. Immunohistochemical Staining

Tissue samples were cut into 5-µm-thick sections and dried overnight at 37 °C in a 311DS-incubator (Labnet International, Inc., Edison, NJ, USA). The samples were stained using a fully automated Leica BOND-MAX staining robot (Leica Microsystems, Wetzlar, Germany) and a horseradish peroxidase-labeled dextran polymer method (Bond Polymer Refine Detection Kit, Leica). The used antibodies are listed in Table S1 (Supplementary Materials). The staining specificity was confirmed using negative isotype controls. Slides were counterstained with hematoxylin and mounted in Mountex (HistoLab, Gothenburg, Sweden).

### 4.3. Double-Label Immunofluorescence Staining

Slides were incubated, after deparaffinization and antigen retrieval, in 0.5% Triton X-100 for 10 min at RT and then incubated in 10% goat or hoarse normal serum for 1 h at RT. Antibodies and their concentrations are listed in Table S2 (Supplementary Materials). After washes in phosphate-buffered saline, samples were incubated in fluorescein-conjugated secondary antibodies for 1 h at room temperature. Nuclei were counterstained with 4′,6-diamidino-2-phenylindole (DAPI) for 10 min at room temperature. Slides were mounted in ProLong^®^ Diamond Antifade Mountant (Thermo Fisher Scientific, Waltham, MA, USA). The specificity of each staining was confirmed with staining controls.

### 4.4. Cell Lines and Culture

The primary human oral keratinocytes (HOKs) isolated form normal oral mucosa (ScienCell, Uppsala, Sweden) were cultured in complete serum-free oral keratinocyte medium (ScienCell, Uppsala, Sweden). The highly invasive human tongue squamous cell carcinoma HSC-3 cell line (JCRB Cell Bank; Osaka National Institute of Health Sciences, Osaka, Japan) and the tongue squamous cell carcinoma SCC-25 cell line (ATCC, Rockville, MD, USA) were cultured in Dulbecco’s modified Eagle medium with Nutrient Mixture F-12 Medium, 10% fetal bovine serum, penicillin–streptomycin, and 0.1% hydrocortisone. Cells were subcultured when they were over 90% confluent, then cells were counted and plated in multi-well cell culture plates (BD Falcon, Lawrence, KS, USA).

### 4.5. Cell Sensitization and Stimulation

Cells were trypsinized, washed, and seeded at a density of 50–100 × 10^3^ cells/mL in multiwall plates. The reagents used to stimulate the HOKs were 1) 50 ng/mL recombinant human lipopolysaccharide-binding protein (LBP; R&D Systems, Inc., Minneapolis, MN, USA) combined with 5 µg/mL *Escherichia coli* ultrapure lipopolysaccharide (LPS-EB; Invivogen, San Diego, CA, USA); 2) 100 ng/mL recombinant human tumor necrosis factor-α (TNF-α); 3) 100 ng/mL recombinant-human interferon-γ (IFN-γ); both reagents were purchased from R&D Systems Inc. (Minneapolis, MN, USA); 4) 100 μM histamine ≥97.0% (Sigma-Aldrich, St. Louis, MO, USA). The cells were stimulated for 8 h, unless otherwise stated. To investigate the potential effect of the activation of H4R on hBD-2 signaling, the HOKs were incubated with 1 µM specific H4R-agonist HST-10 (*N*-(3-(1H-imidazol-4-yl) propyl)-2-cyclohexylacetamide) or 1 µM inverse agonist ST-1007 (both were kindly provided by Professor Holger Stark) for 8 h before adding treatment. Such ligands were previously utilized and showed effective influence on H4R signaling [17,52,53]. Cells were then stimulated with different inflammatory and bacterial mediators as above. Concentrations and time slots were optimized through pilot experiments.

### 4.6. Purification of Total RNA

The total RNA from OLP patients and healthy participants was purified using a Maxwell^®^ 16 Low Elution Volume RNA-FFPE Kit and AS2000 Maxwell^®^ 16 Instrument (firmware 4.97; Promega Corp, Madison, WI, USA) according to the manufacturer’s instructions. Total cellular RNA from cultured HOKs and OTSCC-derived cells was purified using a RNeasy Mini-Kit (Qiagen, Düsseldorf, Germany).

### 4.7. Droplet-Digital Polymerase Chain Reaction (ddPCR) Analysis

Droplet-digital PCR is a highly-sensitive novel technology that allows specific amplification of nucleic-acid targets and their absolute quantification without need of establishment of a standard curve. Droplet-digital PCR is proven to have a low enough detection limit to detect a few positive molecules and rare target mutations [54]. Reactions were performed in appropriate volumes using 20 μL of reaction mix that contained 10 μL of QX200™ EvaGreen^®^ ddPCR™ Supermix (Bio-Rad Laboratories), 2 μL of complementary DNA (cDNA), and 1 μL of primers. Samples were loaded into a droplet generator cartridge and transferred into the supplied cartridges. Seventy microliters of Droplet-Generation Oil (for EvaGreen) was added into the parallel wells of the cartridge, followed by the individual droplet generation, facilitated by placing the cartridge into the droplet generator (QX200™ Droplet-Generator). Following droplet generation, we pipetted 40 μL of droplets into the columns of a 96-well PCR plate, which was sealed with a supplied foil in PX1-PCR Plate Sealer instrument (Bio-Rad, Hercules, CA, USA). The plate was then loaded into a T100 Thermal Cycler (Bio-Rad), and the annealing temperature was set to 60 °C. The sealed plate was then transferred into the droplet reader for detection of completed PCR reactions in droplets, which measured the fraction of positive droplets and calculated the amount of a template per droplet based on a Poisson distribution, with precision estimated at a 95% confidence interval (CI) for each droplet. The data were analyzed using and QX200™ Droplet Digital™ PCR Systems (Bio-Rad Laboratories) according to the manufacturer’s instructions. The QuantaSoft version 1.7.4.0917 software (Bio-Rad Laboratories) was used for data analysis.

### 4.8. Quantitative Real-Time Polymerase Chain Reaction

Quantitative real-time polymerase chain reaction (qRT-PCR) was performed using 2 µL of first-strand cDNA and 250 nmol/L primers in iQ SYBR^®^ Green supermix or SsoAdvanced Universal SYBR^®^ Green Supermix (BioRad Laboratories Inc., Hercules, CA, USA). Human primer sequences are listed in Table S3 (Supplementary Materials).

### 4.9. Imaging and Statistical Analysis

A Leica DM6000 microscope with a Leica DFC365-FX camera (Leica Microsystems, Wetzlar, Germany) was used. The staining intensity of each sample was analyzed using the ImageJ2 software. Data analysis was performed using GraphPad Prism version 6 (GraphPad Software, La Jolla, CA, USA). The Shapiro–Wilk test was used to assess the normality of data. The paired Student’s *t*-test, Mann–Whitney U test, and Kruskal–Wallis followed by Dunn’s multiple comparisons test were applied where appropriate. Data are presented as means ± standard deviation. Statistical significance was set at *p*-values ≤0.05. Significance was indicated as * *p* ≤ 0.05, ** *p* ≤ 0.01, and *** *p* ≤ 0.0001.

## Supplementary Materials

Supplementary Materials can be found at https://www.mdpi.com/1422-0067/20/7/1780/s1.

## Abbreviations

hBD-2	Human β-defensin 2
HXR	Histamine HX receptor
OTSCC	Oral tongue squamous cell carcinoma
OLP	Oral lichen planus
HOKs	Human oral keratinocytes
MC	Mast cell
LPS	Lipopolysaccharides
LBP	Lipopolysaccharide-binding protein
MrgX2	Mas-related gene X2
FFPE	Formalin-fixed paraffin-embedded
