# Supplementary material for: Human β-Defensin 2 Expression in Oral Epithelium: Potential Therapeutic Targets in Oral Lichen Planus
Source: Int J Mol Sci. 2019 Apr 10;20(7):1780. doi: 10.3390/ijms20071780 (PMC6479702; doi:10.3390/ijms20071780)
Supplement: Supplementary file 1 [file ijms-20-01780-s001.pdf]

Table S1. Primary antibodies used for immunohistochemistry

Antibody Name and Clonality	Source	Working Concentration	Other Information
Rabbit anti-human, polyclonal, β-Defensin 2	Bioss, Woburn, MA, USA	5 µg/ml	• Immunogen: 34-64/64<br>• Isotype: IgG
Rabbit anti-human, polyclonal, Histamine H1 Receptor	LifeSpan Biosciences Inc., Seattle, WA, USA	1 µg/ml	• Immunogen: Synthetic 17 amino acid peptide from the 2nd extracellular domain.<br>• Isotype: IgG
Rabbit anti-human, polyclonal, Histamine H2 Receptor	LifeSpan Biosciences Inc., Seattle, WA, USA	1 µg/ml	• Immunogen: Synthetic 16 amino acid peptide from the 3rd extracellular domain.<br>• Isotype: IgG
Rabbit anti-human, polyclonal, Histamine H3 Receptor	LifeSpan Biosciences Inc., Seattle, WA, USA	1 µg/ml	• Immunogen: Synthetic peptide of human H3R.<br>• Isotype: IgG
Rabbit anti-human, polyclonal, Histamine H4 Receptor	LifeSpan Biosciences Inc., Seattle, WA, USA	1 µg/ml	• Immunogen: Synthetic 18 amino acid peptide from the 1st cytoplasmic domain.<br>• Isotype: IgG

Table S2. Primary antibodies used for double-labelling immunofluorescence staining

Antibody Name and Clonality	Source	Working Concentration	Other Information
Rabbit anti-human, polyclonal, beta defensin 2	Bioss, Woburn, MA, USA	5 µg/ml	• Immunogen: 34-64/64<br>• Isotype: IgG
Mouse anti-human, monoclonal, CD4	Dako Cytomation, Glostrup, Denmark	3 µg/ml	• Immunogen: Recombinant protein corresponding to the external domain of the CD4 molecule.<br>• Isotype: IgG1k<br>• Clone: 4B12
Mouse anti-human, monoclonal, CD8	Dako Cytomation, Glostrup, Denmark	1.5 µg/ml	• Immunogen: Synthetic peptide corresponding to the 13 C-terminal amino acids of cytoplasmic domain of human CD8 coupled to thyroglobulin.<br>• Isotype: IgG1<br>• Clone: C8/144B
Mouse anti-human, monoclonal, CD163	Leica Biosystems, Newcastle, UK	10 µg/ml	• Immunogen: recombinant protein corresponding to domains 1 to 4 of the N-terminal region of the CD163 molecule<br>• Isotype: IgG1<br>• Clone: 10D6
Mouse anti-human, monoclonal, mast cell chymase	Thermo Fisher Scientific, MA, USA	5 µg/ml	• Immunogen: Purified human skin chymase<br>• Isotype: IgG1<br>• Clone: CC1
Rabbit anti-human, polyclonal, NFkB p65	Abcam, Cambridge, United Kingdom	1 µg/ml	• Immunogen: Synthetic peptide corresponding to Human NF-kB p65 amino acid 500 to the C-terminus conjugated to Keyhole Limpet Haemocyanin.<br>• Isotype: IgG
Rabbit, anti-human, polyclonal, p-STAT1 (Ser727)	Cell Signaling Technology, Inc. Danvers, MA, USA		• Immunogen: synthetic phosphopeptide corresponding to

	residues surrounding Ser727 of human Stat1.
	• Isotype: IgG

Table S3. List of the human primers for PCR analysis

Gene	Forward	Reverse
hBD-2	5′-ATCAGCCATGAGGGTCTTGT-3′	5′-GAGACCACAGGTGCCAATTT-3′
TNF-α	5′-CTTTGGAGTGATCGGCCCC-3′	5′-GGTTATCTCTCAGCTCCACGC-3′
NF-kB	5′-CCAGACCAACAACAACCCCT-3′	5′-TCACTCGGCAGATCTTGAGC-3′
STAT1	5′-AGTCTGGCGGCTGAATTTCG-3′	5′-GATCACCACAACGGGCAGAG-3′
GAPDH	5′-AAGGTCATCCCTGAGCTG-3′	5′-TGCTGTAGCCAAATTCGTTG-3′
RPLP0	5′-GGCGACCTGGAAGTCCAACT-3′	5′-CCATCAGCACCACAGCCTTC-3′

HBD-2: Human Beta-defensin 2; TNF-α: Tumor necrosis factor alpha; NF-kB: Nuclear factor kappa-light-chain-enhancer of activated B cells; STAT1: Signal transducer and activator of transcription 1; GAPDH: Glyceraldehyde-3-phosphate dehydrogenase; RPLP0: Ribosomal Protein Lateral Stalk Subunit P0.
